# PIP5K1α promotes myogenic differentiation via AKT activation and calcium release

**DOI:** 10.1186/s13287-018-0770-z

**Published:** 2018-02-09

**Authors:** Xiaofan Chen, Jun Wan, Bo Yu, Yarui Diao, Wei Zhang

**Affiliations:** 1 Shenzhen Key Laboratory for Translational Medicine of Dermatology, Biomedical Research Institute, Shenzhen Peking University—the Hong Kong University of Science and Technology Medical Center, Lianhua Road 1120, Shenzhen, 518036 Guangdong Province China; 2 Shenzhen Key Laboratory for Neuronal Structural Biology, Biomedical Research Institute, Shenzhen Peking University—the Hong Kong University of Science and Technology Medical Center, Shenzhen, China; 3 Department of Dermatology, Peking University Shenzhen Hospital, Shenzhen, 518036 Guangdong Province China; 4 Ludwig Institute for Cancer Research, 9500 Gilman Drive, La Jolla, CA 92093 USA

**Keywords:** PIP5K1α, Myogenic differentiation, AKT activation, Calcium release

## Abstract

**Background:**

Skeletal muscle satellite cell-derived myoblasts are mainly responsible for postnatal muscle growth and injury-induced regeneration. Many intracellular signaling pathways are essential for myogenic differentiation, while a number of kinases are involved in this modulation process. Type I phosphatidylinositol 4-phosphate 5-kinase (PIP5KI) was identified as one of the key kinases involved in myogenic differentiation, but the underlying molecular mechanism is still unclear.

**Methods:**

PIP5K1α was quantified by quantitative reverse transcriptase PCR and western blot assay. Expression levels of myogenin and myosin heavy chain, which showed significant downregulation in PIP5K1α siRNA-mediated knockdown cells in western blot analysis, were confirmed by immunostaining. Phosphatidylinositol 4,5-bisphosphate in PIP5K1α siRNA-mediated knockdown cells was also measured by the PI(4,5)P2 Mass ELISA Kit. C2C12 cells were overexpressed with different forms of AKT, followed by western blot analysis on myogenin and myosin heavy chain, which reveals their function in myogenic differentiation. FLIPR assays are used to test the release of calcium in PIP5K1α siRNA-mediated knockdown cells after histamine or bradykinin treatment. Statistical significances between groups were determined by two-tailed Student’s *t* test.

**Results:**

Since PIP5K1α was the major form in skeletal muscle, knockdown of PIP5K1α consistently inhibited myogenic differentiation while overexpression of PIP5K1α promoted differentiation and rescued the inhibitory effect of the siRNA. PIP5K1α was found to be required for AKT activation and calcium release, both of which were important for skeletal muscle differentiation.

**Conclusions:**

Taken together, these results suggest that PIP5K1α is an important regulator in myoblast differentiation.

## Background

Adult mammalian skeletal muscle could induce a rapid and extensive regeneration in response to severe damage. This muscle repair process occurs through the activation of muscle satellite cells quiescent in the basal lamina and the muscle fiber membrane of normal muscles. The activated satellite cells will move outside the basal lamina and differentiate to accelerate the muscle repair. The differentiation of skeletal muscle is required by the myogenic regulatory genes. The myogenic regulatory factors (MRFs), a family of basic helix–loop–helix (bHLH) transcription factors, consist of myogenic differentiation antigen (MYOD) [1], myogenic factor 5 (MYF5) [2], myogenin [3], and MRFs (MRF4) [4–6]. Another group of muscle regulatory transcription factors belong to the myocyte enhancer factor-2 (MEF2) family. There are four MEF2 genes in mammals, including MEF2A, MEF2B, MEF2C, and MEF2D [7–11]. Protein kinases are also key regulators of signal transduction essential for myogenic differentiation, such as the phosphatidylinositol 3-kinase (PI3K)/Akt and the p38 mitogen-activated protein kinase (MAPK)-mediated pathways [12, 13].

Type I phosphatidylinositol 4-phosphate 5-kinase (PIP5KI) is a kinase critical in synthesizing phosphatidylinositol 4,5-bisphosphate (PIP2) through phosphorylating phosphatidylinositol-4-phosphate (PI4P). PIP2 is a substrate of phospholipase C (PLC), which generates the lipid second messengers diacylglycerol (DAG) and inositol 1,4,5-triphosphate (IP3) [14]. DAG activates protein kinase C and IP3 increases the intracellular calcium level by releasing Ca^2+^ from the endoplasmic reticulum. PIP2 can also be phosphorylated by PI3K to generate PIP3, which is another lipid second messenger involved in cell growth, survival, and apoptosis [15]. In addition, PIP2 can act as a second messenger in many cellular processes such as cell migration, adhesion, and division [16, 17]. Thus, PIP5K1 essentially regulates these processes by modulating the production of the multifunctional lipid messenger PIP2.

In mammalians, three isoforms of PIP5K1 have been identified as PIP5K1α, PIP5K1β, and PIP5K1γ [18–20]. In this study, PIP5K1α was considered the major isoform of PIP5K1 in skeletal muscle and required for myogenic differentiation. PIP5K1α was upregulated during myoblast differentiation, while knockdown of PIP5K1α inhibited C2C12 cell differentiation. PIP5K1α promoted myoblast differentiation by regulating the PIP2-mediated AKT pathway and cytoplasmic calcium release. Together, our work shows that PIP5K1α promoted myogenic differentiation via the activation of AKT signaling and modulation of the cytoplasmic calcium level.

## Methods

### Cell culture

C2C12 cells (ATCC, Manassas, VA, USA) were maintained in growth medium (GM; Dulbecco’s modified Eagle’s medium (DMEM) supplemented with 20% fetal bovine serum (FBS), 100 U/ml penicillin, and 100 μg/ml streptomycin) in a 37 °C incubator with 5% CO_2_. To induce differentiation, cells were grown in differentiation medium (DM; DMEM with 2% horse serum).

### Preparation of mouse primary myoblasts

The mice limb muscles were isolated and incubated with 0.1% Pronase in DMEM at 37 °C for 1 hour. After centrifuge at 1500 rpm for 5 min the supernatant was removed, while the pellet was resuspended in 10 ml DMEM and passed through a 40-μm filter to remove muscle debris. The cells were collected by centrifugation, resuspended in 10 ml growth media (Ham’s F-10 medium with 20% FBS and 5 ng/ml beta-fibroblast growth factor (β-FGF)), and transferred to noncoated plates to allow fibroblasts to attach. The floating cells were then transferred to 2% Matrigel-coated (BD Biosciences) plates to facilitate attachment of myoblasts. The growth medium was changed after 24 hours. Myoblasts were trypsinized and transferred to a new Matrigel-coated plate for the following experiments.

### siRNA and plasmid transfection

For siRNA transfection, cells were plated into 12-well plates. For each well, 100 nM siRNA was added with the Lipofectamine RNAiMAX (Invitrogen, Carlsbad, CA, USA) according to the instructions. The sequences of siRNA were listed as follows: PIP5K1α#1, AGAAGUGGGUGGCGUGAAU; and PIP5K1α#2, TCAGAAAGAACGAGAGAAA. For plasmid transfection, cells were plated into 12-well plates and plasmids were added with the Lipofectmine Plus reagents (Invitrogen) according to the instructions.

### Extraction of PI(4,5)P2 from cells and measurement

After siRNA treatment, cells were collected with 1 ml ice-cold 0.5 M TCA and incubated on ice for 5 min. After centrifuge, the pellet was washed twice with 1 ml of 5% TCA/1 mM EDTA. Neutral lipids were extracted with 1 ml MeOH:CHCl_3_ (2:1). Then acidic lipids were extracted with 750 μl MeOH:CHCl_3_:12 N HCl (80:40:1). The supernatant was transferred to a new 2-ml centrifuge tube, and 250 μl CHCl_3_ and 450 μl of 0.1 N HCl added. After vortex and centrifuge, the organic phase were collected into a clean 1.5-ml vial and dried in a vacuum dryer. The measurement of PI(4,5)P2 from cells is a 96-well ELISA assay for detection and quantification of PI(4,5)P2 according to the instructions of the PI(4,5)P2 Mass ELISA Kit (Echelon, USA).

### Antibodies, immunostaining, and western blotting

Anti-myogenin and anti-PIP5K1α were purchased from Santa Cruz Biotechnology, Inc. (Santa Cruz, CA, USA), anti-GAPDH was purchased from Ambion (Austin, TX, USA), anti-phospho-AKT (T308/S473) and anti-total-AKT were purchased from Cell Signaling (Danvers, MA, USA), and anti-MHC was purchased from the Developmental Studies Hybridoma Bank (Iowa City, IA, USA). For immunostaining, C2C12 cells were plated on six-well plates with glass coverslips. After cotransfection with siRNA for 24-hour and then 48-hour treatment of differential medium, cells were fixed in 4% paraformaldehyde for 15 min and permeabilized by 0.2% Triton X-100 for 15 min. Cells were rinsed in PBS, blocked in 5% BSA for 1 hour, and then incubated with anti-myogenin and anti-MHC antibody (1:500) overnight. Cells were washed three times in PBS and incubated with fluorescein-conjugated secondary antibodies (Jackson ImmunoResearch Laboratories Inc., West Grove, PA, USA) for 1 hour. Then 100 ng/ml of DAPI was added for another 10 min to stain the nuclei. After three washes in PBS, the coverslips were mounted and cells visualized using an Olympus IX70 fluorescence microscope. Western blot analysis was performed according to procedures described previously [21].

### RNA preparation and quantitative real-time PCR

Total RNA from cells was extracted with TRIzol reagent (Invitrogen). Following the manufacturer’s instructions, the expression level of PIP5K1 was detected by SYBR Green-based qRT-PCR with FastStart Universal SYBR Green Master mix (Roche). The sequences of primers are listed as follows: PIP5K1A forward primer, 5′-CTGATGATTACTTGTACTCCCT-3′; PIP5K1A reverse primer, 5′-CATCACTGGACACATAGAAG-3′; PIP5K1B forward primer, 5′-AGTTCCTGCAGAAGCTGCTG-3′; PIP5K1B reverse primer, 5′-CCTGACTGCATGCAATACAG-3′; PIP5K1C forward primer, 5′-GAGTTCATCATCAAGACTGT-3′; PIP5K1C reverse primer, 5′-GTTGAGATTCATGTAGTAGC-3′; GAPDH forward primer, 5′-TGCACCACCAACTGCTTAGC-3′; and GAPDH reverse primer, 5′-GGCATGGACTGTGGTCATGAG-3′.

### FLIPR assay

After siRNA transfection, the C2C12 cells were seeded into 96-well microtiter plates and incubated with 300 μM bradykinin for 16 hours. On the following day, the cells were labeled with 100 μl labeling medium containing Opti-MEM/Hanks’ balanced salt solution (HBSS), 2.5% FBS, 20 mM HEPES (pH 7.4), 2.5 nM probenecid, and 2 μM Fluo-4 at 37 °C for 60 min. Then 70 μl 3× drugs were prepared and aliquotted into the corresponding wells in the V-well drug plate. Changes in fluorescence were detected in the FLIPR 96 (Molecular Devices, Sunnyvale, CA, USA).

### Statistical analysis

Statistical significances between groups were determined by two-tailed Student’s *t* test. *p* < 0.05 was considered statistically significant.

## Results

### PIP5K1α was upregulated during myoblast differentiation

**Fig. 1:**
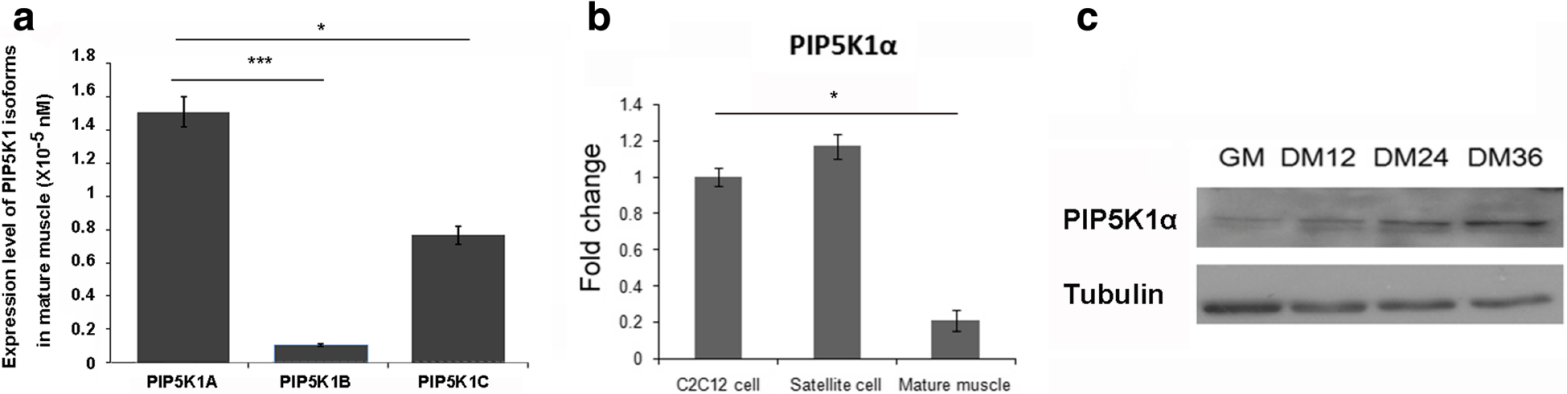
PIP5K1α is the major isoform of PIP5KI in skeletal muscle. **a** Expression of three PIP5KI isoforms in mature muscles. **b** Expression of PIP5K1α in C2C12 cells, primary myoblasts (Satellite cell), and mature muscles. **c** Protein levels of PIP5K1α in C2C12 cells harvested at different time points during differentiation. Data presented as mean ± SD. *p < 0.05, ***p < 0.001. PIP5KI phosphatidylinositol 4-phosphate 5-kinase, GM growth medium, DM differentiation medium

To explore the potential role of PIP5K1 isoforms in myogenic differentiation, we first examined their expression patterns. As shown in Fig. 1a, PIP5K1α was dominantly expressed in skeletal muscle and accumulated in C2C12 and primary myoblast cells (Fig. 1b). Moreover, the protein level of PIP5K1α was gradually increased during C2C12 differentiation (Fig. 1c). This suggested PIP5K1α might have a role in muscle differentiation.

### Knockdown of PIP5K1α inhibited C2C12 differentiation

**Fig. 2:**
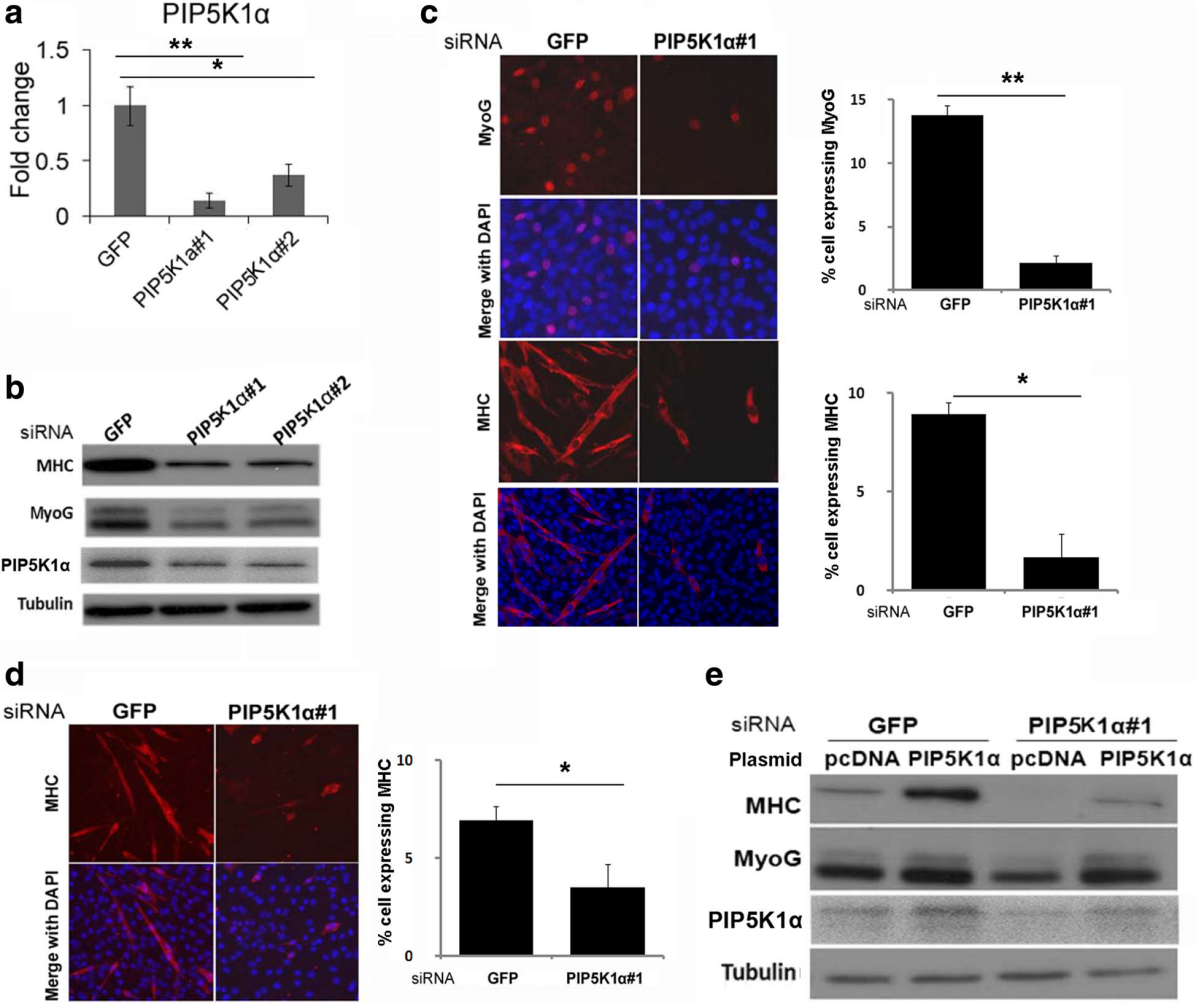
PIP5K1α is required for myoblast differentiation. **a** Knockdown efficiency of PIP5K1α siRNA measured by real-time PCR. **b** C2C12 cells transfected with PIP5K1α siRNA or GFP siRNA as a control; 24 hours after transfection, cells were induced to differentiate for 48 hours. Cells harvested for western blot analysis. **c** C2C12 cells transfected with PIP5K1α siRNA or GFP siRNA as a control. After transfection for 24 hours, cells were induced to differentiate for 48 hours. Cells fixed for immunostaining with myogenin and MHC antibodies. **d** Primary muscle satellite cells isolated from mouse skeletal muscle and cultured for 3 days. Cells transfected with siRNA, and fixed for immunostaining with MHC antibody. **e** C2C12 cells cotransfected with siRNA and siRNA-resistant PIP5K1α cDNA plasmids or empty vector. After transfection for 48 hours, cells subjected to western blot analysis. Data presented as mean ± SD. *p < 0.05, **p < 0.01. PIP5KI phosphatidylinositol 4-phosphate 5-kinase, MHC myosin heavy chain, MyoG myogenin, DAPI 4′,6-diamidino-2-phenylindole, siRNA small interfering RNA. *GFP* Green Florescent Protein

To further reveal the role of PIP5K1α in C2C12 differentiation, the RNAi technique was introduced. Two siRNAs were designed to target different regions of PIP5K1α, both of which inhibited the expression of PIP5K1α efficiently at the mRNA and protein levels (Fig. 2a, b). Cells transfected with these siRNAs showed decreased expression of both myogenin and myosin heavy chain (MHC), which were early and late differentiation markers respectively (Fig. 2b). Similar results were obtained by immunostaining (Fig. 2c). MHC-positive multinucleated myotubes were reduced in the cells transfected with the PIP5K1α-targeting siRNA (Fig. 2c). Consistently, knockdown of PIP5K1α also inhibited the primary myoblast differentiation (Fig. 2d). Thus, the results demonstrated that PIP5K1α was required for myogenic differentiation. As shown in Fig. 2e, the efficient inhibited expression of PIP5K1α by its targeting siRNA was rescued by overexpression of PIP5K1α. As a result, the effect of siRNA on muscle differentiation was partially rescued by the overexpression of PIP5K1α, which indicated that the changed differentiation status was not due to offtarget effects of siRNA. Furthermore, overexpression of PIP5K1α increased expression of myogenin and MHC, which suggested that PIP5K1α promoted myogenic differentiation (Fig. 2e).

### PIP5K1α promoted myoblast differentiation by regulating the AKT pathway

**Fig. 3:**
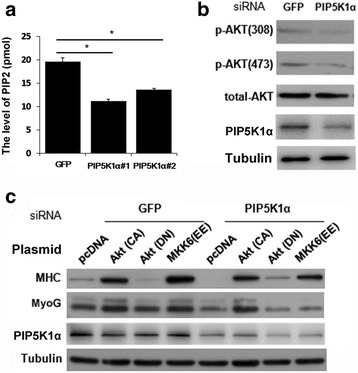
PIP5K1α regulates myoblast differentiation through the AKT pathway. **a** C2C12 cells transfected with PIP5K1α siRNA show decreased production of PIP2. Data presented as mean ± SD. *p < 0.05. **b** C2C12 cells transfected with PIP5K1α siRNA show decreased phosphorylation of AKT. **c** C2C12 cells cotransfected with siRNA and AKT CA, AKT DN, or MKK6 S207E, T211E constitutively active mutant (MKK6 EE) plasmids. Cells harvested after 24-hour transfection and 48-hour treatment of differential medium, followed by western blot analysis. PIP2 phosphatidylinositol 4,5-bisphosphate, PIP5KI phosphatidylinositol 4-phosphate 5-kinase, CA constitutively active, DN dominant-negative, MHC myosin heavy chain, MyoG myogenin, siRNA small interfering RNA. *MKK6(EE)* MKK6 S207E, T211E constitutively active mutant, *GFP* Green Fluorescent Protein

The intracellular PIP2 of mammalian cell is mainly catalyzed by PIP5K1 and subsequently affects the PI3K/AKT pathway. After knockdown of PIP5K1α, C2C12 cells suffered a significant decrease in the production of PIP2 (Fig. 3a). The activation of AKT was important to myogenic differentiation [12]. Knockdown of PIP5K1α suppressed the phosphorylation of AKT, which suggested the AKT pathway might play a role in the promyogenic effect of PIP5K1α (Fig. 3b). To examine whether PIP5K1α-mediated myogenic differentiation was specifically affected by the activation of AKT, PIP5K1α siRNA was cotransfected with plasmids carrying a constitutively active AKT (AKT CA) form or a dominant-negative AKT (AKT DN) form. As shown in Fig. 3c, only overexpression of constitutively active AKT promoted C2C12 cell differentiation and rescued the myogenic inhibition of PIP5K1α targeting siRNA. As we know, the MKK6 (EE) form can activate the p38 pathway which is also required for myogenic differentiation [22]. Although MKK6 (EE) could also strongly promote differentiation, it failed to rescue the myogenic inhibition of PIP5K1α-targeting siRNA.

### PIP5K1α regulated PIP2-mediated cytoplasmic calcium release

**Fig. 4:**
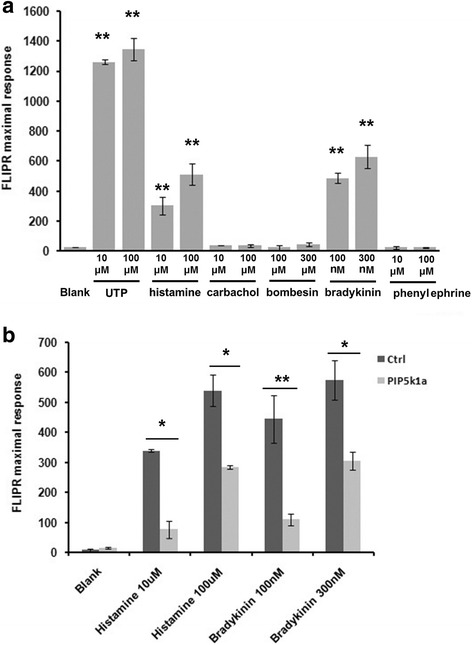
PIP5K1α regulates PIP2-mediated cytoplasmic calcium release. **a** C2C12 cells treated with drugs targeting different G protein receptors followed by FLIPR® Calcium Assay (FLIPR) assays. **b** C2C12 cells transfected with siRNA and treated with histamine or bradykinin for another 16 hours. Cells then subjected to FLIPR assays. Data presented as mean ± SD. *p < 0.05, **p < 0.01. PIP5KI phosphatidylinositol 4-phosphate 5-kinase, Ctrl control. *FLIPR* FLIPR® Calcium Assay

PIP2 can be hydrolyzed by PLC and converted to DAG and inositol triphosphate (IP3), which are essential for the intracellular calcium level. Cytoplasmic calcium has been reported important in myogenic differentiation [23, 24]. Several drugs targeting different G protein receptors were tested. Only histamine and bradykinin were found to induce the release of calcium in C2C12 cells (Fig. 4a). Interestingly, histamine and bradykinin receptors were sensitive to PIP2 [25]. To investigate whether PIP5K1α can affect the cytoplasmic calcium level, C2C12 cells transfected with PIP5K1α siRNA were treated with histamine or bradykinin, which exhibited an obvious defect of cytoplasmic calcium release (Fig. 4b).

## Discussion

In our study, we first found that PIP5K1α was gradually increased during myogenic differentiation, which suggests its role in myogenesis. Calcium signaling is important for differentiation-dependent gene expression. Keratinocyte differentiation involves an intricate pathway involving an acute and sustained rise of the intracellular free calcium level [26]. PIP5K1α activation is also an important step in calcium-induced keratinocyte differentiation [27], which is consistent with its role in myogenic differentiation through regulating the intracellular free calcium level. Interestingly, the expression level of PIP5K1α was much lower in mature muscle than that in satellite cells and C2C12 cells. Similar developmental patterns in the expression of MyoD and myogenin, myogenic transcriptional regulatory proteins, were found during myogenesis [28]. This suggested that these factors play distinct roles in the control of myogenesis.

**Fig. 5:**
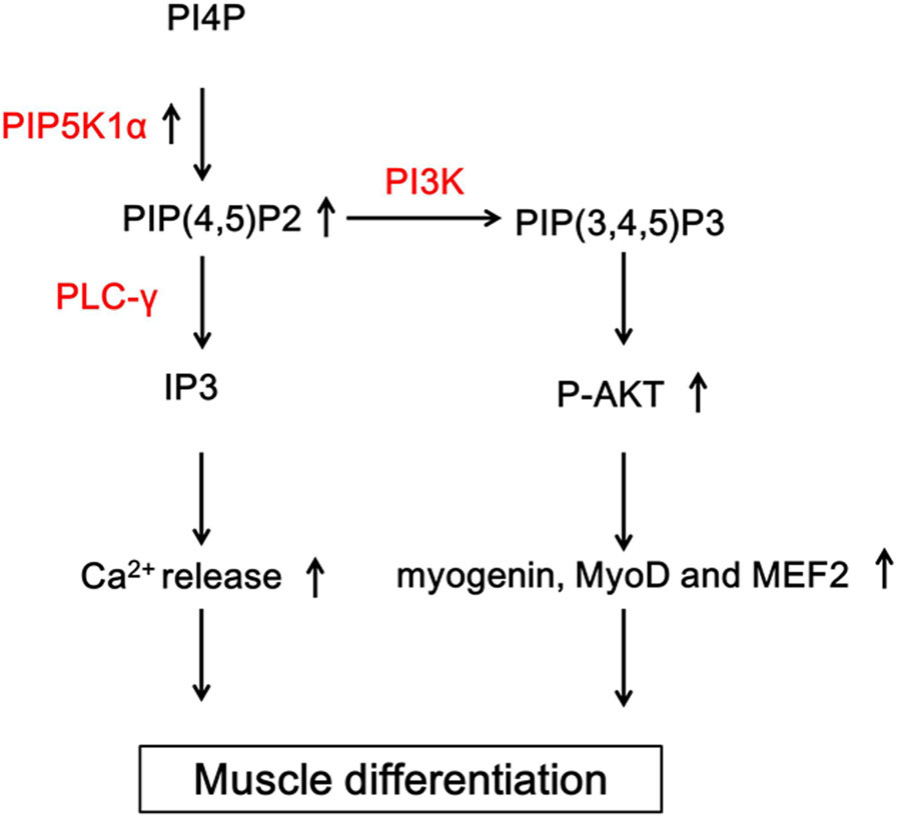
PIP5K1α regulates myogenic differentiation through two pathways. Activated PIP5K1α synthesizes more PIP2 to provide sufficient substrate for PI3K and PLC-γ. On one hand, PIP2 levels are constantly maintained at a high level to enable generation of IP3 required for the increase in intracellular calcium, which is necessary for initiating muscle differentiation. On the other, increased intracellular PIP2 of mammalian cell catalyzed by PIP5K1α subsequently affects the PI3K/AKT pathway. Activation of AKT was also important to myogenic differentiation. PI4P phosphatidylinositol-4-phosphate, PIP5KI phosphatidylinositol 4-phosphate 5-kinase, PI3K phosphatidylinositol 3-kinase, PLC phospholipase C, IP3 inositol 1,4,5-triphosphate, MEF2 myocyte enhancer factor-2. *PIP(4,5)P2* Phosphatidylinositol 4,5-bisphosphate, *PIP(3,4,5)P3* Phosphatidylinositol (3,4,5)-trisphosphate

Our studies have investigated the role of PIP5K1α in inducing muscle differentiation via the activation of AKT signaling and modulation of the cytoplasmic calcium level (Fig. 5). PIP5K1α is the key regulator for the production of PIP2, which is important for various signal transductions in mammalian cells. The activated PIP5K1 synthesizes more PIP2 in the plasma membrane to provide sufficient substrate for PI3K and PLC-γ, which is an essential regulatory step to sustain the activation of PI3K and PLC-γ in muscle. As a result, PIP2 levels are constantly maintained at a high level to enable the generation of IP3 required for the increase in intracellular calcium necessary for initiating muscle differentiation.

The crucial role of Akt (also known as protein kinase B) has been demonstrated previously in the proliferation, survival, differentiation, and viability of muscle cells [29]. Akt regulates protein expression through the mammalian target of rapamycin (mTOR) signaling pathway, which plays an important role in muscle cell differentiation. Furthermore, the IGF/PI3K/Akt signaling pathway has been shown to stimulate myogenic differentiation by inducing the expression of myogenin, MyoD, and MEF2 in normal myogenic cells [12, 30]. Therefore, we hypothesize that PIP5K1α promotes myogenic differentiation by regulating Akt signaling through increasing PIP2 production. Further understanding of the regulation of PIP5K1α will be obtained through exploring more downstream genes regulated by PIP5K1α.

## Conclusions

Our results demonstrate PIP5K1α is required for the activation of AKT signaling and modulation of the cytoplasmic calcium level, which indicates that PIP5K1α regulates myogenic differentiation through multiple pathways.

## Abbreviations

PI3K: Phosphatidylinositol 3-kinase
DAG: Diacylglycerol
IP3: Inositol triphosphate
PI4P: Phosphatidylinositol-4-phosphate
PIP2: Phosphatidylinositol 4,5-bisphosphate
